# Supplementary material for: REDD1 promotes obesity-induced metabolic dysfunction via atypical NF-κB activation
Source: Nat Commun. 2022 Oct 22;13:6303. doi: 10.1038/s41467-022-34110-1 (PMC9588012; doi:10.1038/s41467-022-34110-1)
Supplement: Supplementary file 1 — Supplementary Information [file 41467_2022_34110_MOESM1_ESM.pdf]

SUPPLEMENTAL INFORMATION

REDD1 promotes obesity-induced metabolic dysfunction via atypical NF-κB activation

Supplementary Table 1. List of qRT-PCR primers

Genes	Forward primer sequence	Reverse primer sequence
Ccl2	GCTGCTACTCATTCACCAGCA	ACAGACCTCTCTCTTGAGCTTGG
Tnfa	CGACGTGGAACTGGCAGAA	AGTTCAGTAGACAGAAGAGCGTGGT
Il1b	GTTGACGGACCCCAAAAGAT	TGATACTGCCTGCCTGAAGC
Il6	AGAGGAGACTTCACAGAGGATACCA	TTGCCATTGCACAACTCTTTTC
Pparg	GAGATTCTCCTGTTGACCCA	TCTTCCATCACGGAGAGGT
Cebpa	CTTCTACGAGGTGGAGCC	TCTATAGACGTCTCGTGCTC
aP2	ACAAGGAAAGTGGCAGGC	TTCACCTTCCTGTCGTCTG
Acc	GCCTCTTCCTGACAAACGAG	TGACTGCCGAAACATCTCTG
Fasn	CCCTTGATGAAGAGGGATCA	ACTCCACAGGTGGGAACAAG
Scd-1	TGCCCCTGCGGATCTT	GCCCATTCGTACACGTCATT
G6pc	ATCTGGTTCCATCTTAAAGAGAC	TGCCACCCAGAGGAGATTGATG
Pck1	GACTTCTCTGCCAAGGTCATCCA	GCCATCGCAGATGTGGATATA
Fbp1	TATCAGCACCCTGACCCGCTTC	CGATACCATAGAGCTGTGCGAT
Socs3	GGACCAAGAACCTACGCATCCA	CACCAGCTTGAGTACACAGTCG
Gapdh	CAAAATGGTGAAGGTCGGTG	GAGGTCAATGAAGGGGTCGT

Supplementary Table 2. List of PCR primers for Chip assay

# of NF-kB site	Forward primer sequence	Reverse primer sequence	Product size (bp)
#1,2	GCCTTGGCCATCAACCCAA	CCTGAAGTAGGCAGGAAGTCTC	228
#2	TGGGGAAAGGCTTTTCTTCCTTA	CCACTGCGGCTGACTGAAA	114
#3,4	TGTCAGCCTCCTGCTAATGTC	AGCCGAGAGGAATCTTCAGTC	136
#5	AGACTGAAGATTCCTCTCGGC	CTGGTGTGAGCAGAGGATCG	104
#6	GAGCTACCGAGATTAGTGCC	AACTTGAATCGGGGACTGTG	98

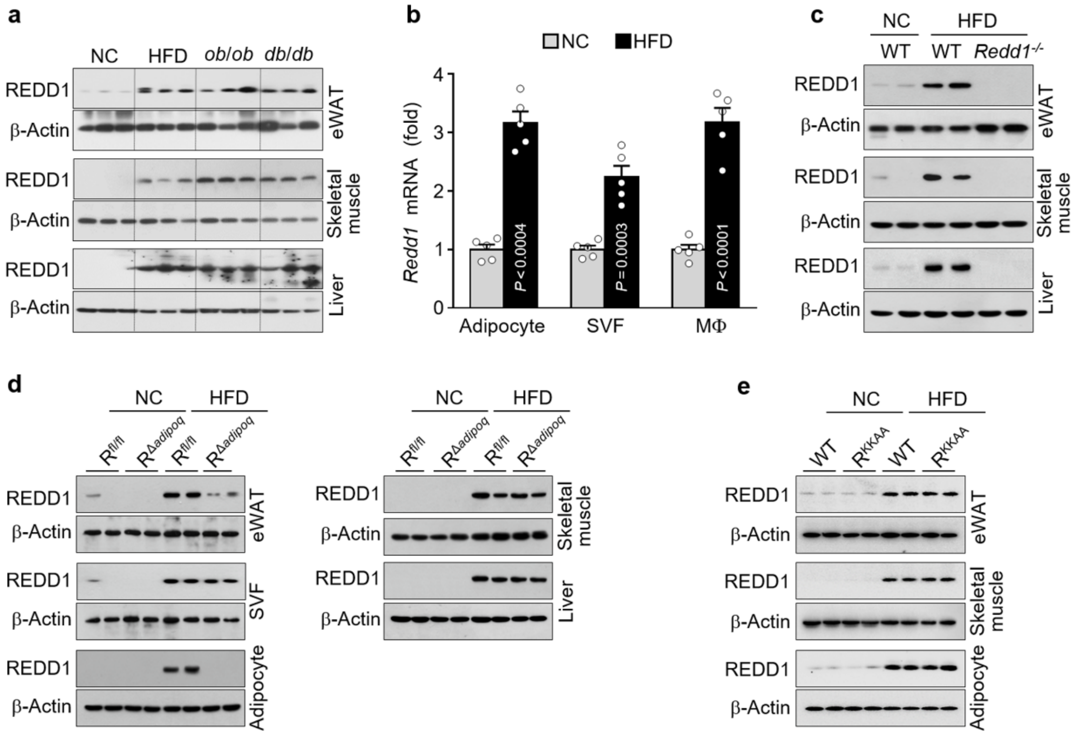

Supplementary Fig. 1. REDD1 expression in obese mice. a, Representative images of western blots showing REDD1 expression in the eWAT, skeletal muscle, and liver of *ob*/*ob*, *db*/*db*, and C57BL/6 mice fed NC or HFD for 16 weeks ($n = 3$). **b**, qRT-PCR-based quantification of *Redd1* in adipocytes, stromal vascular fraction (SVF), and macrophages isolated from eWAT of NC- or HFD-fed C57BL/6 mice ($n = 5$ per group). Statistical significance was calculated using an unpaired two-tailed *t*-test. Bar graphs represent mean ± s.e.m. **c**, Representative western blots for REDD1 expression in eWAT, SVF, skeletal muscles, and liver from NC- or HFD- fed $Redd1^{-/-}$ mice and WT littermates ($n = 3$). **d**, Representative western blots for REDD1 expression in eWAT, SVF, purified adipocytes, skeletal muscle, and liver from NC- or HFD- fed $Redd1^{\Delta adipoq}$ ($R^{\Delta adipoq}$) and $Redd1^{fl/fl}$ ($R^{fl/fl}$) mice ($n = 3$). **e**, Representative western blots for REDD1 expression in various tissues of NC- or HFD- fed $Redd1^{KKAA}$ (R^{KKAA}) mice and their WT littermates ($n = 3$). Source data are provided as a Source Data file.

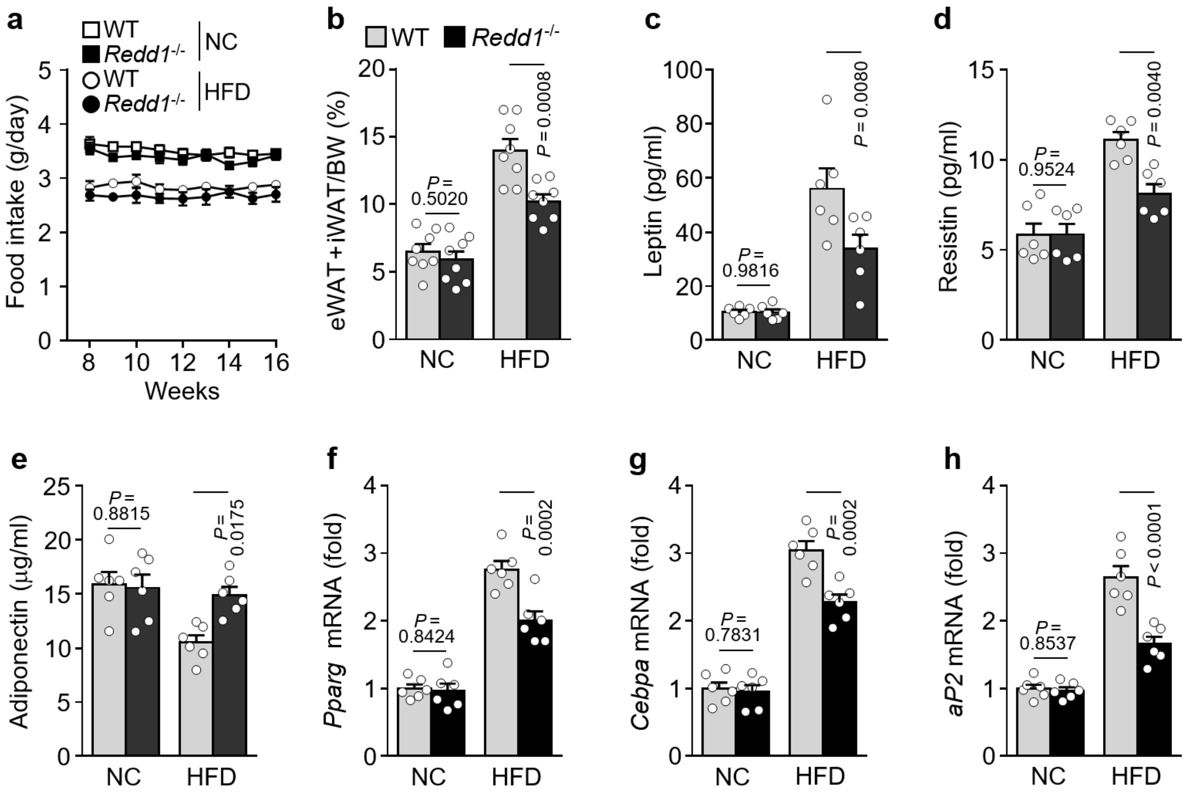

Supplementary Fig. 2. Changes in food intake, normalized fat mass, adipokine production, and adipogenic gene expression in *Redd1*$^{-/-}$ and WT mice. a, Trajectories of daily food intake for *Redd1*$^{-/-}$ mice and WT littermates fed NC or HFD between 8 and 16 weeks (n = 6 per group). **b**, Changes in fat (eWAT + iWAT) mass after normalization for body weight (BW) in mice fed NC or HFD for 16 weeks (n = 8 per group). **c**–**e**, Plasma levels of leptin (**c**), resistin (**d**), and adiponectin (**e**) in mice fed NC or HFD for 16 weeks (n = 6 per group). **f**–**h**, Expression levels of *Pparg* (**f**), *Cebpa* (**g**), and *aP2* (**h**) in eWAT of mice fed NC or HFD for 10 weeks (n = 6 per group). Data are shown as mean ± s.e.m. Statistical significance was calculated using two-way ANOVA followed by the Holm–Sidak post hoc test. Source data are provided as a Source Data file.

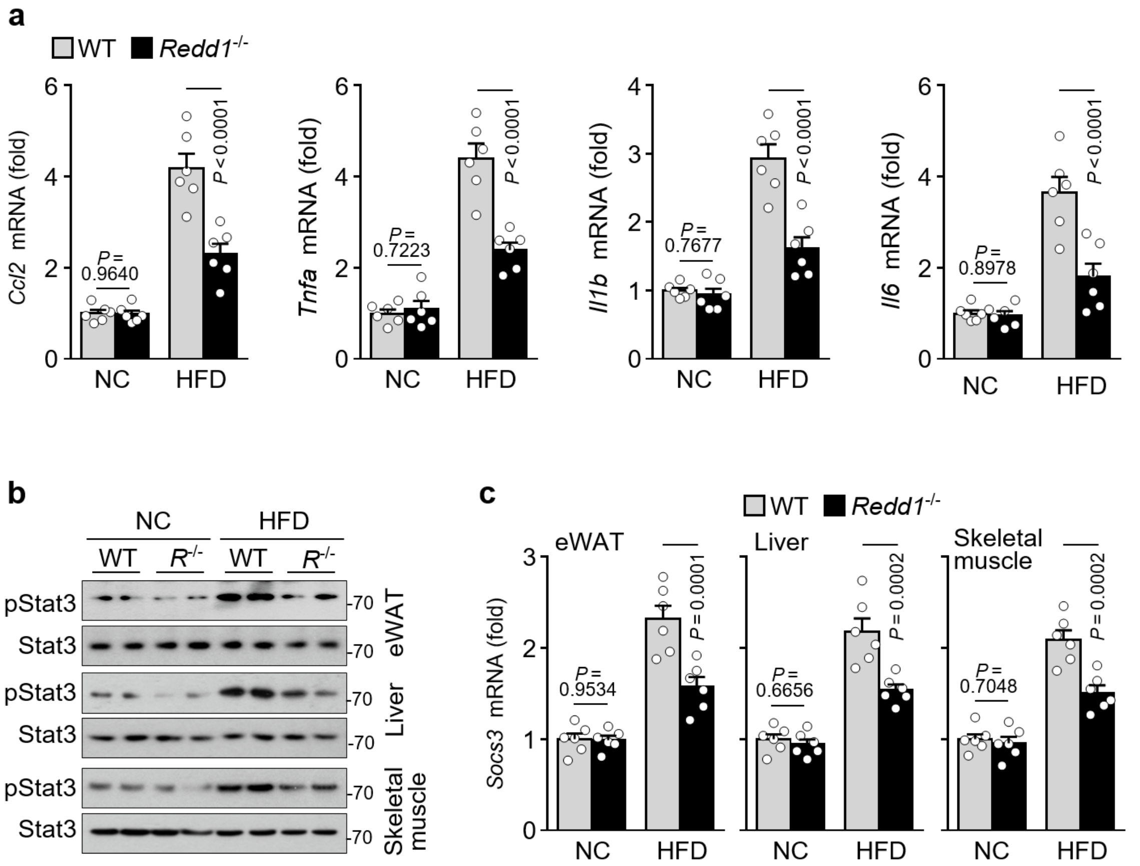

Supplementary Fig. 3. Expression of proinflammatory cytokines and their downstream signaling pathway in *Redd1*$^{-/-}$ and WT mice. a, Expression levels of *Ccl2*, *Tnfa*, *Il1b*, and *Il6* in eWAT of NC- or HFD-fed *Redd1*$^{-/-}$ ($R^{-/-}$) mice and WT littermates (n = 6 per group). **b**, Representative western blots of phosphorylated Stat3 in various tissues of NC- or HFD-fed mice (n = 3). **c**, qRT-PCR-based quantification of *Socs3* levels in various tissues of NC- or HFD-fed mice (n = 6 per group). Data are shown as mean ± s.e.m. Statistical significance was calculated using two-way ANOVA followed by the Holm–Sidak post hoc test. Source data are provided as a Source Data file.

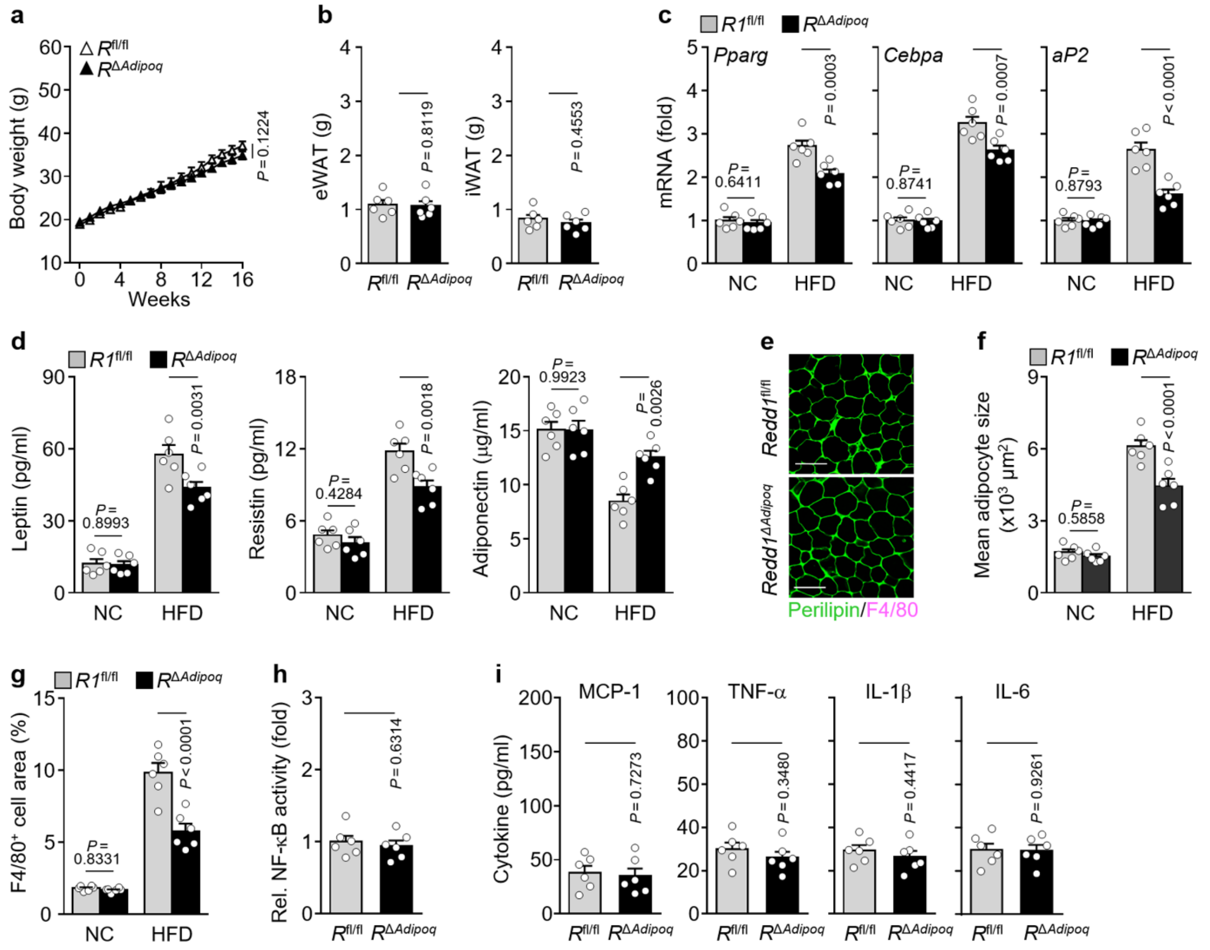

Supplementary Fig. 4. Effects of adipocyte *Redd1* deletion on adipogenesis and inflammation. a, Weight gain over time in *Redd1*$^{\text{fl/fl}}$ (*R*$^{\text{fl/fl}}$) and *Redd1*$^{\Delta Adipoq}$ (*R*$^{\Delta Adipoq}$) mice fed NC for 16 weeks (n = 6 per group). **b**, Measurements for eWAT and iWAT mass in NC-fed mice (n = 6 per group). **c**, Expression levels of *Pparg*, *Cebpa*, and *aP2* in eWAT of mice fed NC or HFD for 10 weeks (n = 6 per group). **d**, Plasma levels of adipokines in mice fed NC or HFD for 16 weeks (n = 6 per group). **e**, Representative images showing perilipin (green) and F4/80 (purple) staining in eWAT of NC-fed mice (n = 6 per group). Scale bar, 100 μm. **f**, Average adipocyte size in eWAT of NC- or HFD-fed mice (n = 6 per group). **g**, Relative area of F4/80-positive cells in eWAT of NC- or HFD-fed mice (n = 6 per group). **h**, NF-κB activity in eWAT from NC-fed mice (n = 6 per group). **i**, Plasma levels of inflammatory cytokines in NC-fed mice (n = 6 per group). Data are shown as mean ± s.e.m. Statistical significance was calculated using an unpaired two-tailed t-test (**a**, **b**, **h**, **i**) and two-way ANOVA followed by the Holm–Sidak post hoc test (**c**, **d**, **f**, **g**). Source data are provided as a Source Data file.

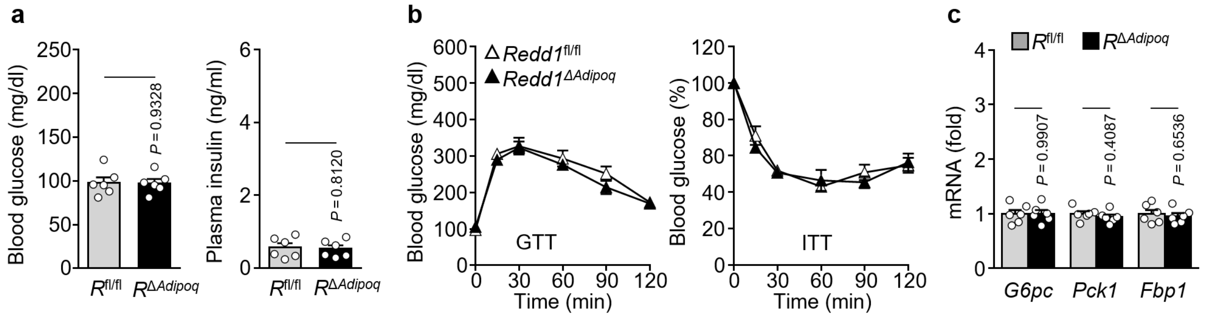

Supplementary Fig. 5. Effects of adipocyte *Redd1* deletion on insulin resistance and glucose metabolism. **a**, Fasting plasma levels of glucose and insulin in NC-fed *Redd1*$^{fl/fl}$ ($R^{fl/fl}$) and *Redd1*$^{\Delta LysM}$ ($R^{\Delta LysM}$) (n = 6 per group). **b**, Assessment of GTT and ITT in NC-fed mice fasting for 12 and 6 h, respectively (n = 6 per group). **c**, Relative expression levels of *G6pc*, *Pck1*, and *Fbp1* in the liver of NC-fed mice (n = 6 per group). Bar graphs represent mean ± s.e.m. Statistical significance was calculated using an unpaired two-tailed *t*-test. Source data are provided as a Source Data file.

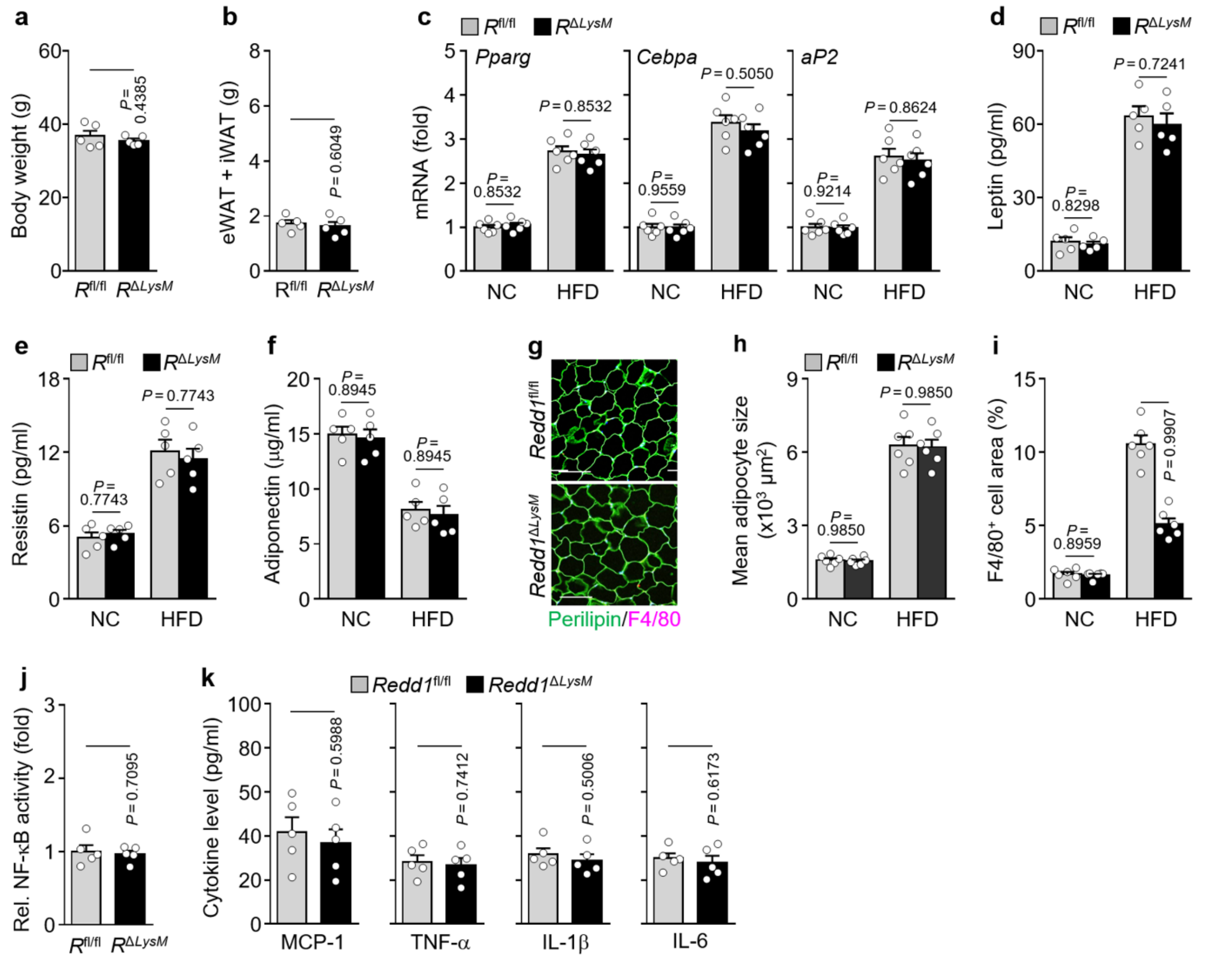

Supplementary Fig. 6. Effects of myeloid *Redd1* deletion on adipogenesis and inflammation. a, Weight gain over time in *Redd1*$^{fl/fl}$ ($R^{fl/fl}$) and *Redd1*$^{\Delta LysM}$ ($R^{\Delta LysM}$) mice fed NC for 16 weeks (*n* = 5 per group). **b**, Measurements for eWAT + iWAT mass in NC-fed mice (*n* = 5 per group). **c**, Expression levels of *Pparg*, *Cebpa*, and *aP2* in eWAT of mice fed NC or HFD for 10 weeks (*n* = 6 per group). **d–f**, Plasma levels of adipokines in mice fed NC or HFD for 16 weeks (*n* = 5 per group). **g**, Representative images showing perilipin (green) and F4/80 (purple) staining in eWAT of NC-fed mice (*n* = 6 per group). Scale bar, 100 µm. **h**, **i**, Average adipocyte size (**h**) and relative area of F4/80-positive cells (**i**) in eWAT of NC- or HFD-fed mice (*n* = 6 per group). **j**, NF-κB activity in eWAT from NC-fed mice (*n* = 5 per group). **k**, Plasma levels of inflammatory cytokines in NC-fed mice (*n* = 5 per group). Data are shown as mean ± s.e.m. Statistical significance was calculated using an unpaired two-tailed *t*-test (**a**, **b**, **j**, **k**) and two-way ANOVA followed by the Holm–Sidak post hoc test (**c–f**, **h**, **i**). Source data are provided as a Source Data file.

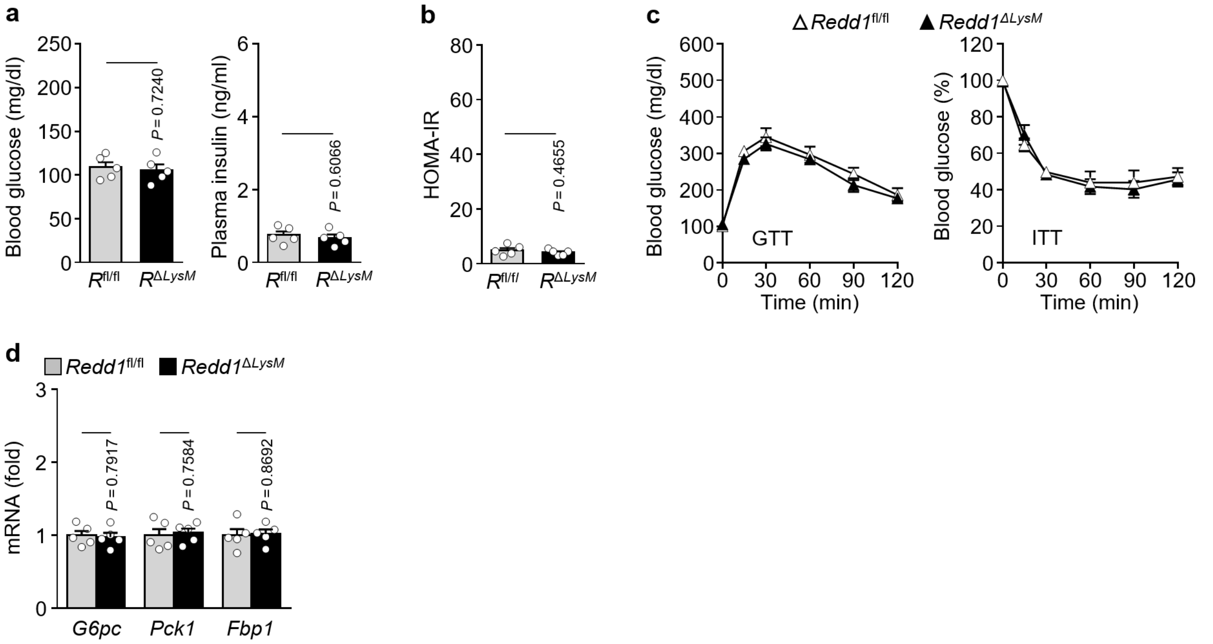

Supplementary Fig. 7. Effects of myeloid *Redd1* deletion on insulin resistance and glucose metabolism. a, Fasting plasma levels of glucose and insulin in NC-fed *Redd1*$^{fl/fl}$ (*R*$^{fl/fl}$) and *Redd1*$^{\Delta LysM}$ (*R*$^{\Delta LysM}$) mice (n = 5 per group). **b**, Calculation of HOMA-IR scores in NC-fed mice (n = 5 per group). **c**, Assessment of GTT and ITT in NC-fed mice fasting for 12 and 6 h, respectively (n = 5 per group). **d**, Expression levels of *G6pc*, *Pck1*, and *Fbp1* in the liver of NC-fed mice (n = 5 per group). Bar graphs represent mean ± s.e.m. Statistical significance was calculated using an unpaired two-tailed *t*-test. Source data are provided as a Source Data file.

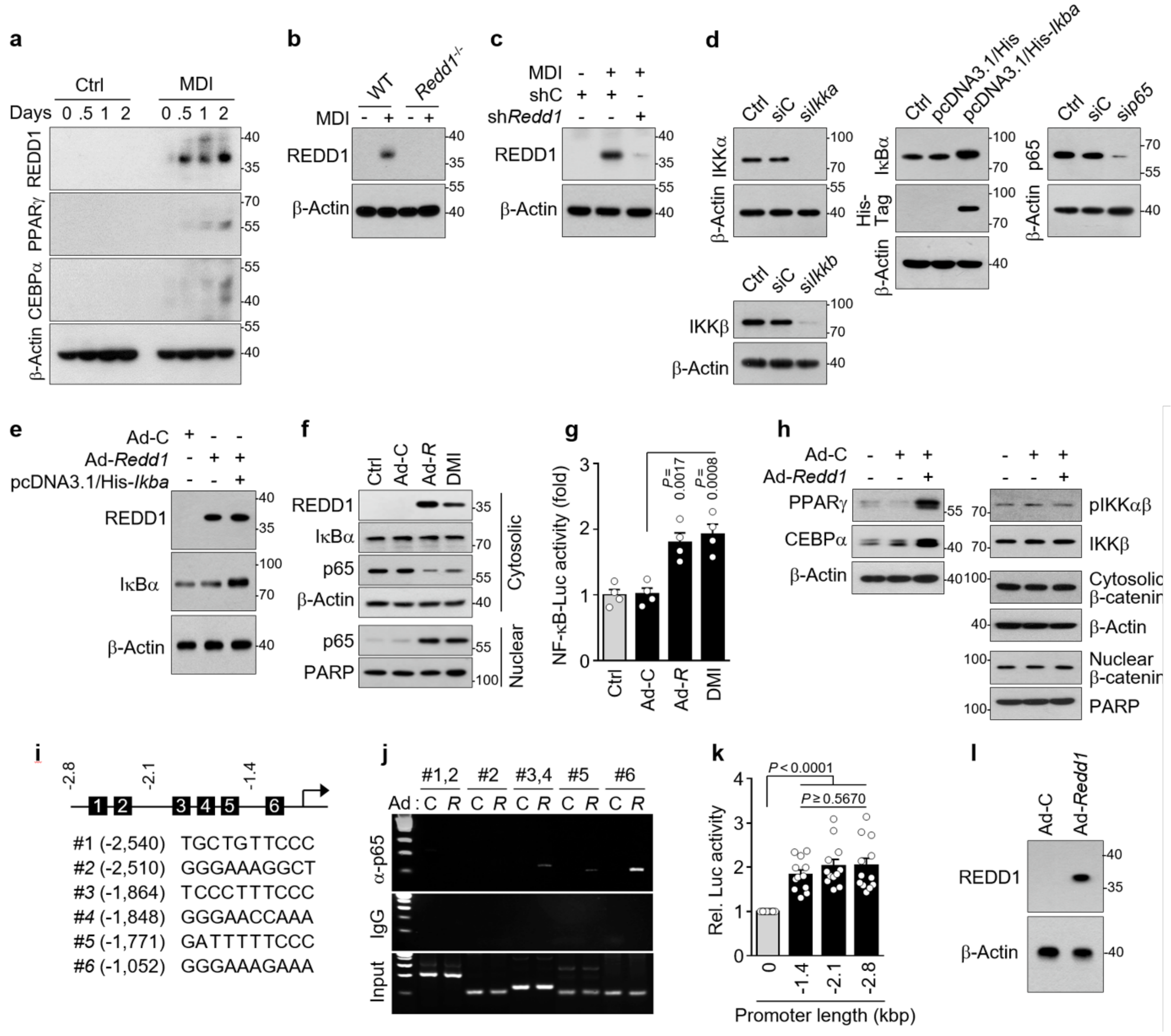

Supplementary Fig. 8. Effects of REDD1 on NF-κB activation and adipogenic gene expression. **a**, Expression of REDD1, PPARγ, and CEBPα in SVF cells cultured in a differentiation medium containing MDI (*n* = 3). **b**, Expression of REDD1 in WT and *Redd1*$^{-/-}$ SVF cells cultured in MDI medium (*n* = 3). **c**, Expression of REDD1 in 3T3-L1 cells exposed to MDI medium following transfection with shControl (shC) and sh*Redd1* (*n* = 3). **d**, Target gene expression in 3T3-L1 cells transfected either with siRNA for control, *Ikka*, *Ikkb*, or NF-κB *p65* or with pcDNA3.1/His-*Ikba* (*n* = 3). **e**, Expression of REDD1 and IκBα in 3T3-L1 cells transfected with pcDNA3.1/His-*Ikba* or infected with control adenovirus (Ad-C) or adenoviral *Redd1* (Ad-*Redd1*) (*n* = 3). **f**, **g**, NF-κB activation (**f**) and NF-κB-Luc activity (**g**) in 3T3-L1 cells infected with Ad-*Redd1* (Ad-*R*) or cultured in MDI medium (*n* = 4). **h**, Levels of PPARγ, CEBPα, phospho-IKKαβ, and cytosolic and nuclear β-catenin in 3T3-L1 cells infected with Ad-C and Ad-*R* (*n* = 3). **i**, Schematic diagram showing six putative NF-κB binding sites and their nucleotide sequences in *Cebpa* promoter. **j**, Chip analysis was performed in Ad-C (C) and Ad-*R(R)*-infected 3T3-L1 cells using specific primers (Supplementary table 2) (*n* = 3). **k**, *Cebpa* promoter-luciferase activity was assayed in 3T3-L1 cells infected with Ad-C or Ad-*R* (*n* = 10). **l**, Expression of REDD1 in mouse peritoneal macrophages infected with Ad-C and Ad-*Redd1* (*n* = 3).

Luciferase activity (**g**, **k**) represent mean ± s.e.m. Statistical significance was calculated using one-way ANOVA followed by the Holm–Sidak post hoc test. Source data are provided as a Source Data file.

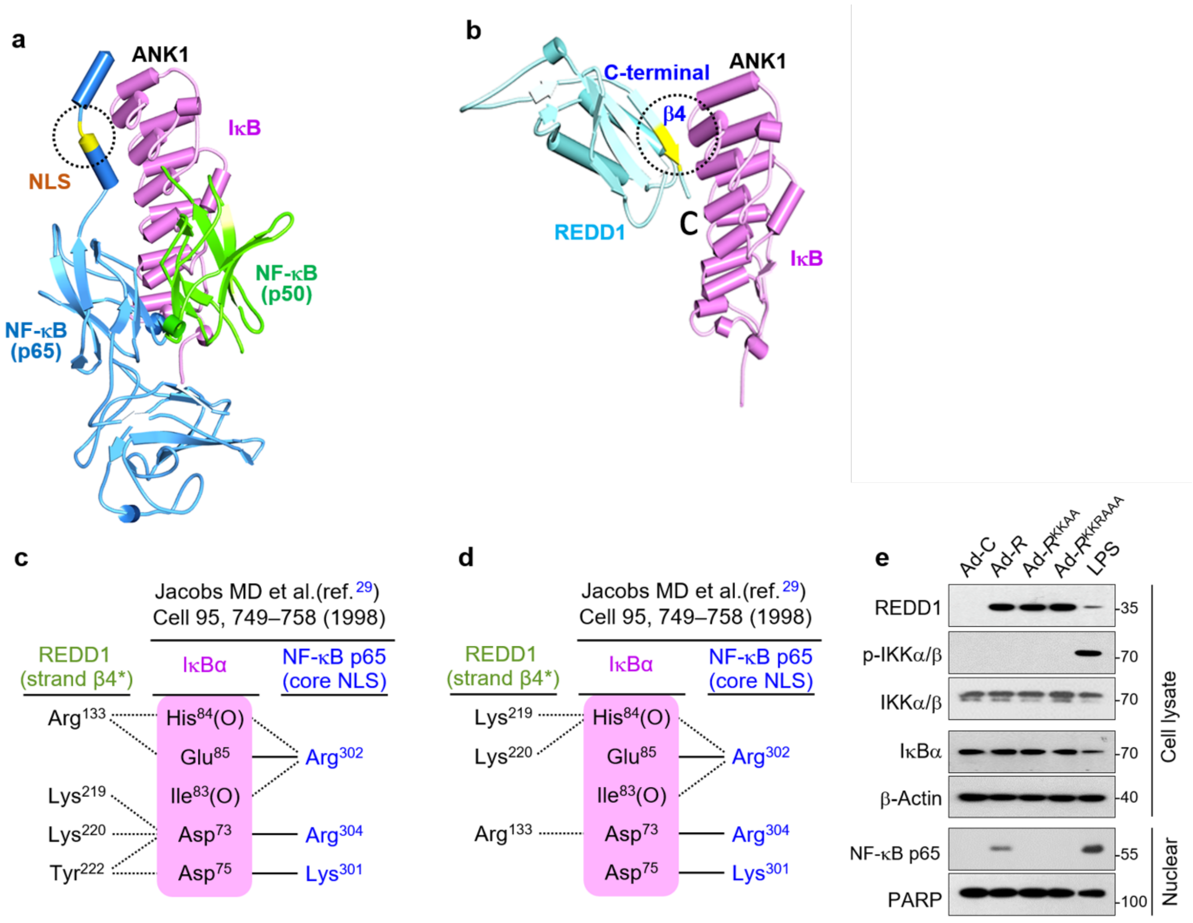

Supplementary Fig. 9. Computational prediction of interaction sites between IκBα and NLS of NF-κB p65 or strand β4 of REDD1. a, **b**, The complex structure of IκBα and NF-κB (p65/p50) in the X-ray crystal structure (**a**, ref.[29]) and the predictive binding conformation of IκBα and REDD1 (**b**) using computational protein-protein docking methods. The dotted circle indicates a key binding site. **c**, **d**, Key residue interactions between IκBα and NF-κB p65 or REDD1 in the protein-protein docking using the HADDOCK (**c**) and HDOCK (**d**) servers, respectively. Solid and dotted lines indicate salt bridges and hydrogen bonds, respectively. **e**, NF-κB activation in 3T3-L1 cells infected with Ad-*Redd1* or its mutants or treated with 100 ng/ml lipopolysaccharide (LPS) as a positive control (n = 3). Source data are provided as a Source Data file.

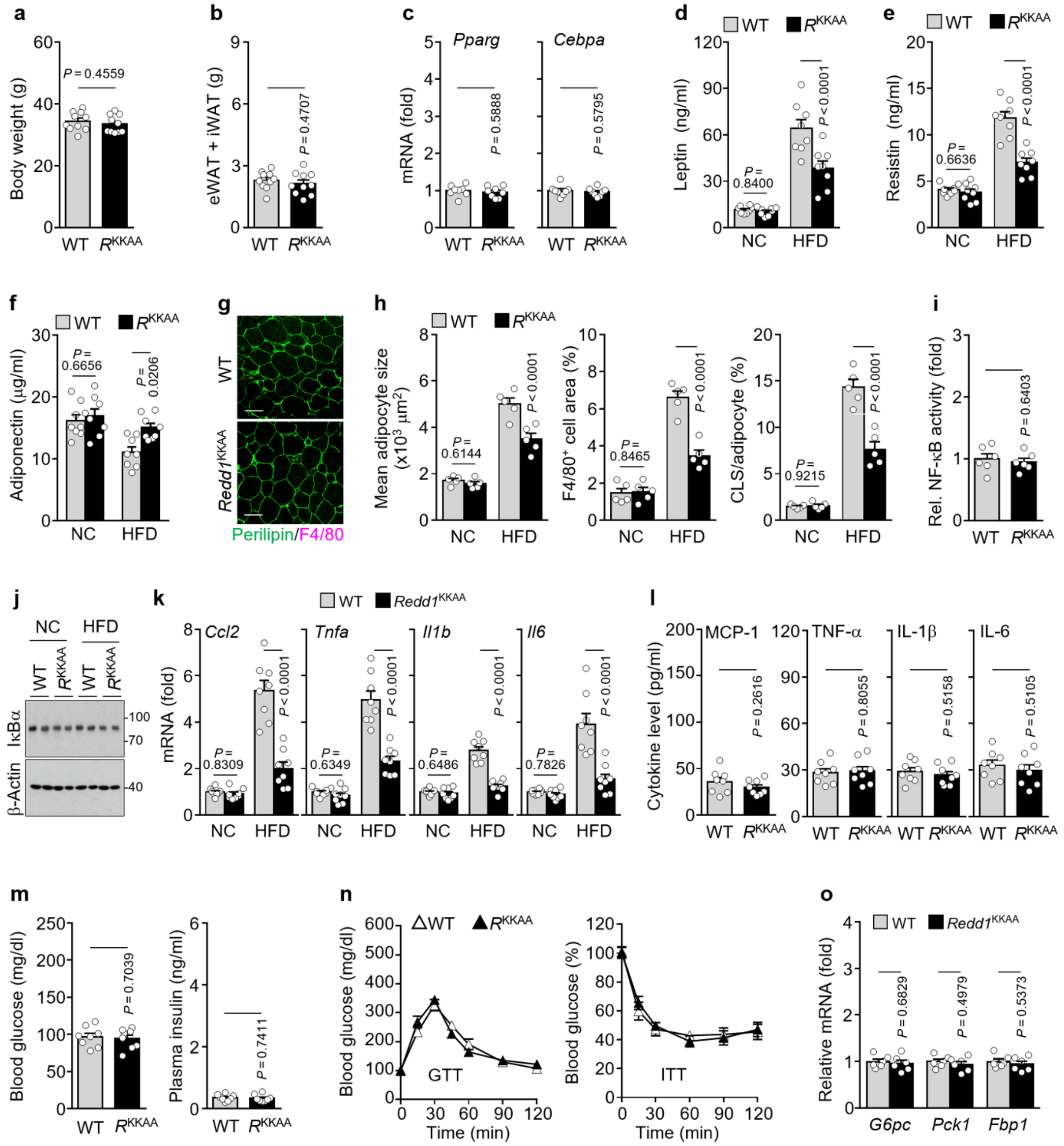

Supplementary Fig. 10. Effects of *Redd1*^{KKAA} on adipogenesis, inflammation, and glucose metabolism. a, Weight gain over time in *Redd1*^{KKAA} (*R*^{KKAA}) mice and their WT littermates fed NC for 16 weeks (*n* = 10 per group). **b**, Measurement for eWAT + iWAT mass in NC-fed mice (*n* = 10 per group). **c**, Expression levels of *Pparg* and *Cebpa* in eWAT of mice fed NC for 10 weeks (*n* = 8 per group). **d–f**, Plasma levels of adipokines in mice fed NC or HFD for 16 weeks (*n* = 8 per group). **g**, Representative images showing perilipin (green) and F4/80 (purple) staining in eWAT of mice fed NC for 16 weeks. Scale bar, 100 µm. **h**, Average adipocyte size, the relative area of F4/80-positive cells, and the relative number of crown-like structures (CLSs) in eWAT of NC- or HFD-fed mice (*n* = 5 per group). **i**, NF-κB activity in eWAT of NC-fed mice (*n* = 6 per group). **j**, Representative western blots for IκBα expression in eWAT of NC- or HFD-fed mice. **k**, Expression levels of cytokine genes of NC- or HFD-fed mice (*n* = 8 per group). **l**, Plasma levels of inflammatory cytokines in NC-fed mice (*n* = 8

per group). **m**, Fasting plasma levels of glucose and insulin in NC-fed mice (*n* = 8 per group). **n**, Assessment of GTT and ITT in NC-fed mice after fasting for 12 and 6 h, respectively (*n* = 6 per group). **o**, Relative expression levels of *G6pc*, *Pck1*, and *Fbp1* in the liver of NC-fed mice (*n* = 6 per group). Data are shown as mean ± s.e.m. Statistical significance was calculated using an unpaired two-tailed *t*-test (**a–c**, **i**, **l**, **m–o**) and two-way ANOVA followed by the Holm–Sidak post hoc test (**d–f**, **h**, **k**). Source data are provided as a Source Data file.

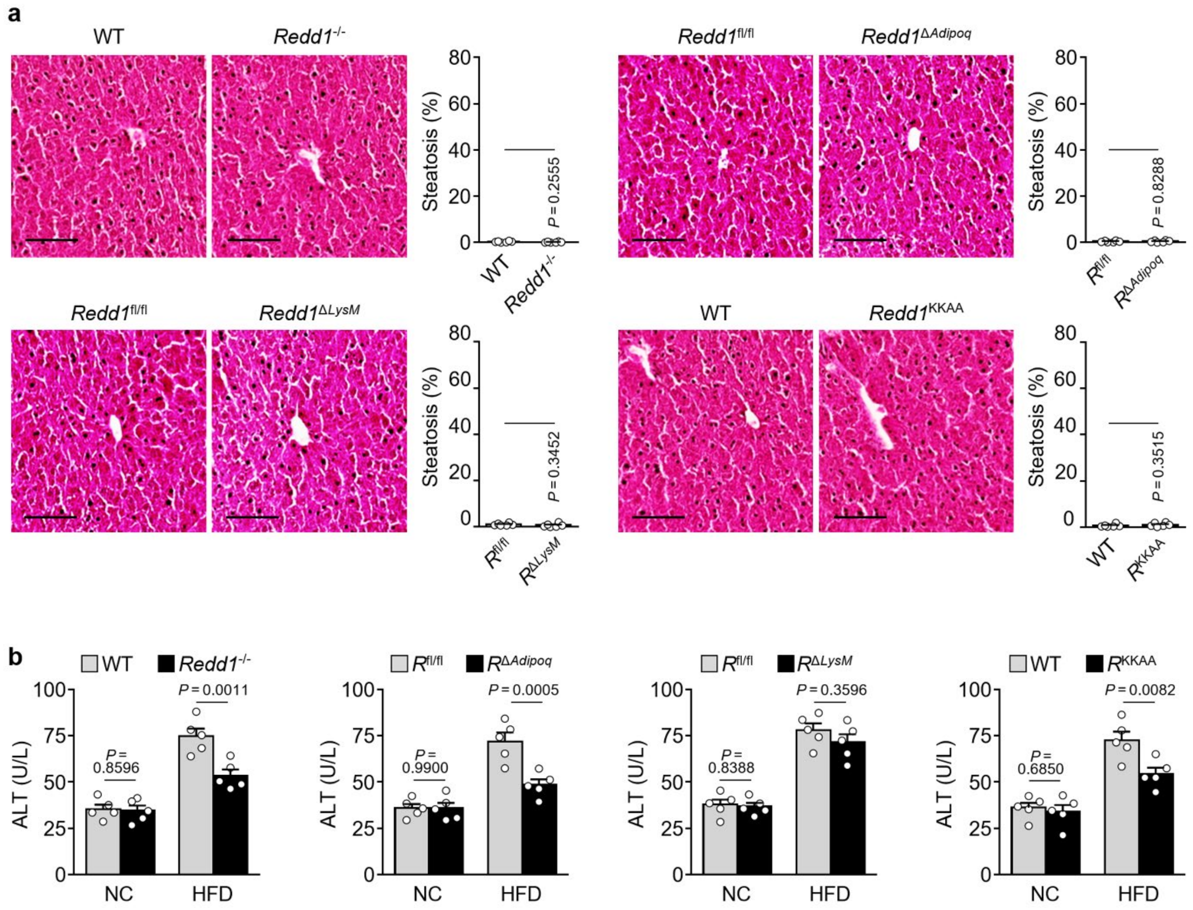

Supplementary Fig. 11. Effects of *Redd1* deletion on hepatic steatosis. **a**, Representative images of H&E-stained liver tissues from NC-fed *Redd1*$^{-/-}$, *Redd1*$^{\Delta Adipoq}$, *Redd1*$^{\Delta LysM}$, *Redd1*^{KKAA}, and control mice, and quantification of hepatic steatosis from H&E-stained liver tissues (*n* = 6 per group). Scale bars, 100 μm. **b**, Plasma levels of ALT in NC- or HFD-fed *Redd1*-deficient and control mice (*n* = 6 per group). Bar graphs represent mean ± s.e.m. Statistical significance was calculated using an unpaired two-tailed *t*-test (**a**) and two-way ANOVA followed by the Holm–Sidak post hoc test (**b**). Source data are provided as a Source Data file.

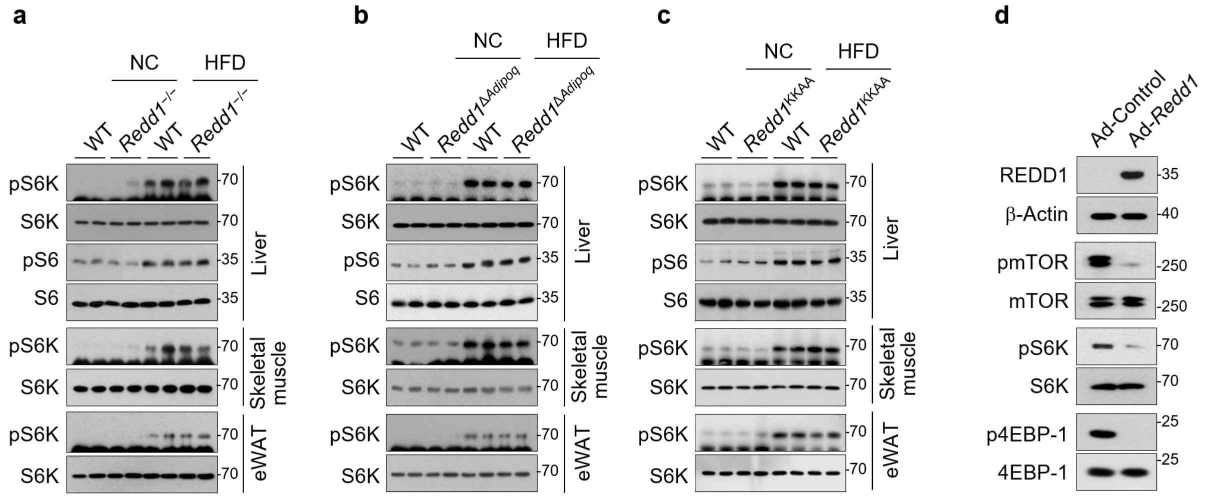

Supplementary Fig. 12. Effects of *Redd1* deletion or overexpression on the mTOCC1 pathway. a–c, Representative western blots for phosphorylation of the downstream mTORC1 effectors S6K and S6 in the liver, skeletal mice, and eWAT of NC- or HFD-fed *Redd1*$^{-/-}$ (**a**), *Redd1*$^{\Delta Adipoq}$ (**b**), *Redd1*^{KKAA} (**c**), and control mice (n = 3). **d**, Representative western blots for mTOR, S6K, and 4EBP-1 phosphorylation in 3T3-L1 cells transfected with Ad-C or Ad-*Redd1* (n = 3). Source data are provided as a Source Data file.

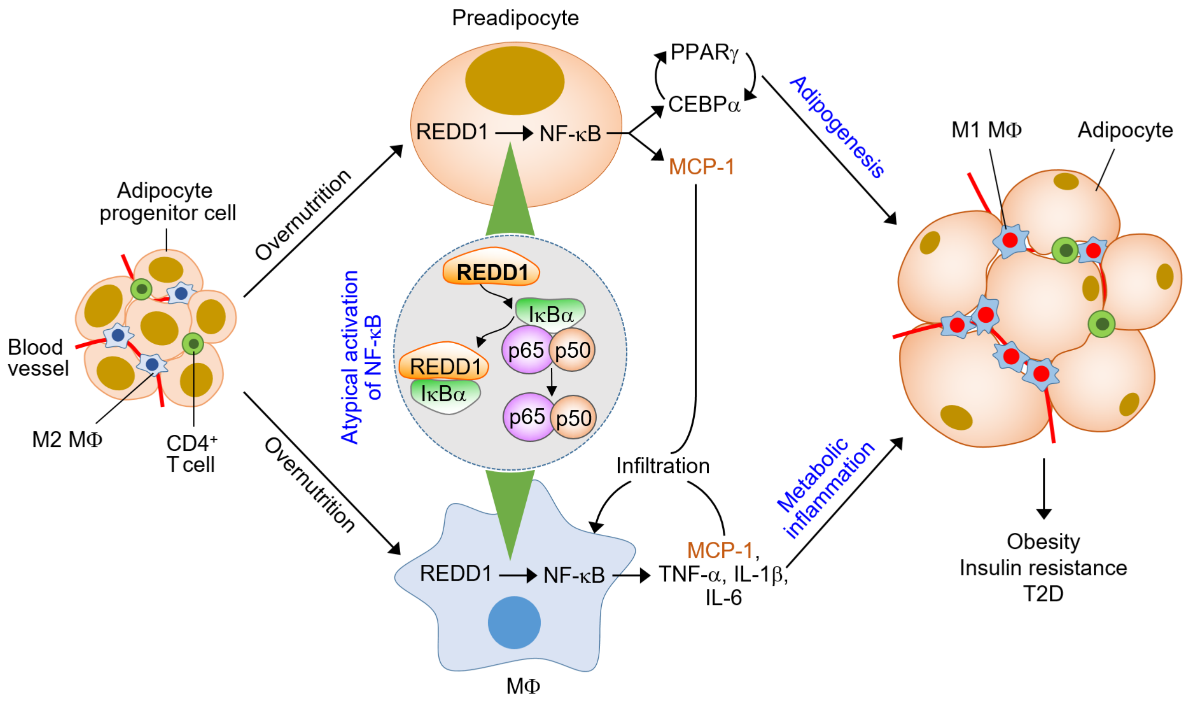

Supplementary Fig. 13. Proposed model for the role of REDD1–NF-κB axis in adipogenesis and metabolic dysregulation. REDD1 is induced or upregulated in adipocytes and macrophages of adipose tissue by overnutrition and binds with and sequesters IκBα away from inactive NF-κB complexes in the cytoplasm. The liberated NF-κB dimers translocate to the nucleus to activate target gene expression. NF-κB activated in adipocytes contributes to adipogenesis and obesity through upregulation of PPARγ and CEBPα and stimulates inflammatory gene expression, including MCP-1 that promotes macrophage infiltration into adipose tissue. In macrophages, NF-κB activation results in meta-inflammation and ultimately insulin resistance and T2D.

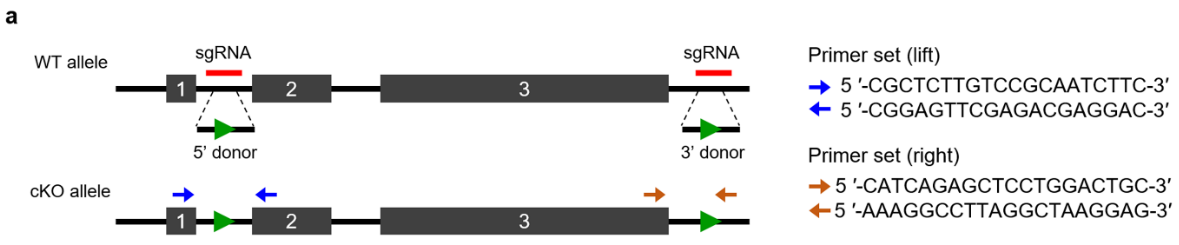

b

Target locus	Target sequence for sgRNA	Donor sequence
5′ (left)	CCTCCCGGGTGTGCACATCTTGT<br>CGGGTGTGCACATCTTGTCGGGG<br>CCCGGGTGTGCACATCTTGTCGG	GTTGGCACCAGTTCGCTCACCCTTCGAGAGGCAGAGCGCTCTTG TCCGCAATCTTCGCTGACCGCGCTAGCTGGTGAGTGTCCCTCCC GGGTGTGCATAACTTCGTATAATGTATGCTATACGAAGTTATACAT CTTGTCGGGGAGTGACCTCGGCTCTTGGGGCCTCAGAGCCCTCA ATCCTGTGCAACGCCGACACTG
3′ (right)	CCTCCCTGGAAGTCTGAGTCTCT<br>CCCTGGAAGTCTGAGTCTCTACA<br>AGTCTGAGTCTCTACAAGGAGGG	GGTCCCTCAGTTCTGCTGAGTGGAGCTACCAGGAGAATGTGCTG GAAAGCACCCGAAGGTGAAAGATGAGTCAGACCTCCCTGGAAGT CTATAACTTCGTATAATGTATGCTATACGAAGTTATGAGTCTCTAC AAGGAGGGTTACCTTGCTCTCCAGGTGAGAAGGCCCAGTCTCCT TAGCCTAAGGCCTTTTCTCTGA
Red, PAM; Green, LoxP sequence		

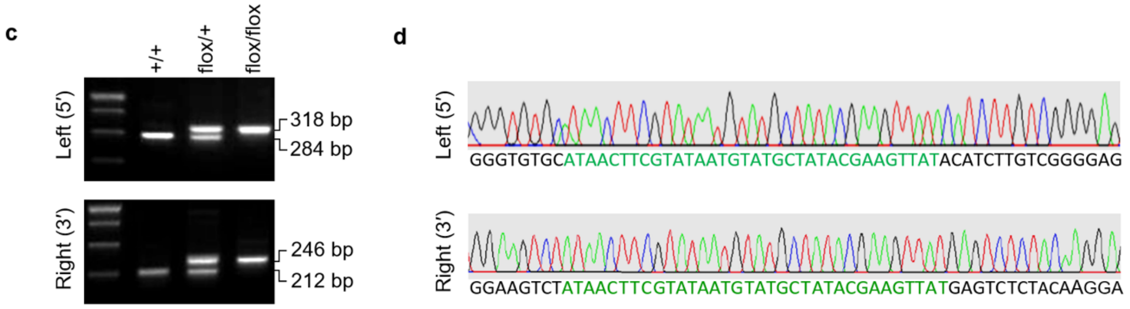

Supplementary Fig. 14. Generation of *Redd1*$^{fl/fl}$ mouse using CRISPR/Cas9-mediated gene editing. **a**, A schematic representation of targeting sites in *Redd1*. PCR primer sets capable of verifying the insertion of loxP into left (5′) and right (3′) positions. **b**, Sequence of sgRNAs and long ssDNA donors containing loxP sequence. **c**, PCR analysis of genomic DNA from WT, *Redd1*$^{fl/+}$, and *Redd1*$^{fl/fl}$ mice (*n* = 3). **d**, Confirmation of genomic DNA sequences of inserted loxP into left (5′) and right (3′) positions. Source data are provided as a Source Data file.

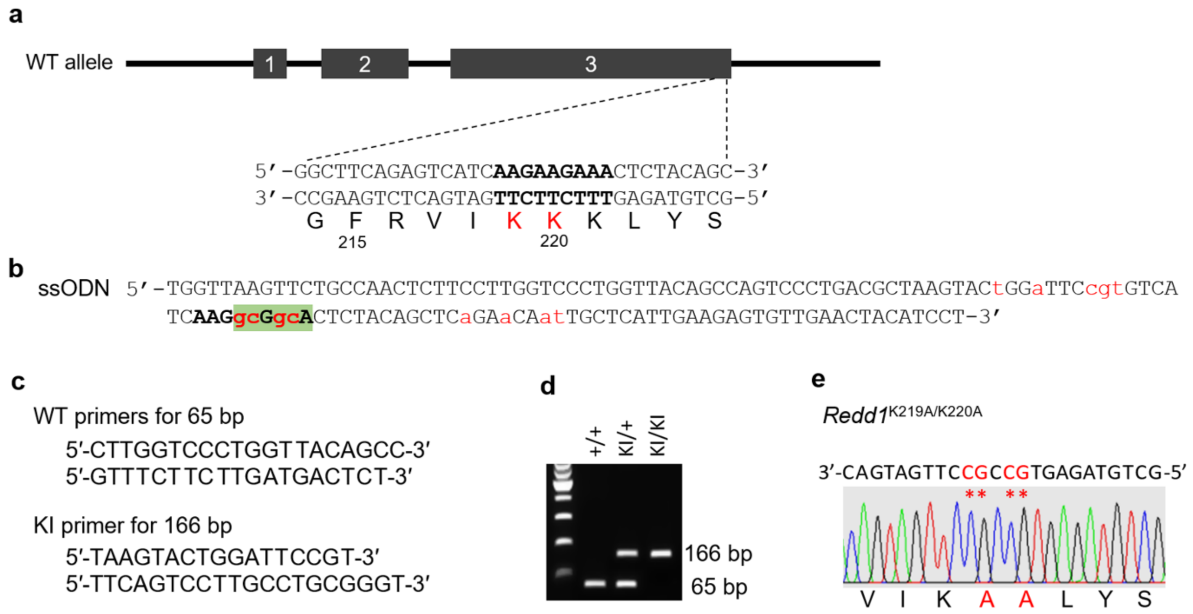

Supplementary Fig. 15. Generation of *Redd1*^{KKAA} mouse using CRISPR/Cas9-mediated gene editing. **a**, A schematic representation of targeting sites (bold sequence) in *Redd1*. **b**, Sequence of ssODN. The 136-bp ssODN donor template shows the Lys^{219}Ala/Lys^{220}Ala mutant sequence in the green box. The silent mutations used to prevent re-cutting by Cas9 are represented as small letters in red. **c**, PCR primers used for detection of WT and knock-in (KI) mutant *Redd1*. **d**, PCR analysis of genomic DNA from WT and *Redd1*^{KKAA} knock-in mice (n = 3). **e**, Confirmation of genomic DNA sequences of *Redd1*^{KKAA} mutation. Asterisks indicate knock-in mutations. Source data are provided as a Source Data file.
